# Dyspnea Induced by Alpha 2-Adrenergic Agonists and Dissociative Anesthetics Combination in Dogs and Cats

**DOI:** 10.3390/ani16071100

**Published:** 2026-04-03

**Authors:** Taehoon Sung, Won-gyun Son, Junghee Yoon, Cheol-yong Hwang, Inhyung Lee

**Affiliations:** Department of Veterinary Clinical Sciences, College of Veterinary Medicine, Seoul National University, Seoul 08826, Republic of Korea; xogns7615@snu.ac.kr (T.S.); carpeego@snu.ac.kr (W.-g.S.); heeyoon@snu.ac.kr (J.Y.); cyhwang@snu.ac.kr (C.-y.H.)

**Keywords:** hypertension, drug combination, juvenile animals, pulmonary hemorrhage, atropine

## Abstract

This case report describes the potential adverse effects of the combination of alpha 2-adrenergic agonists and dissociative anesthetics and discusses its prevention. The cases of 2 dogs and 3 cats, including 4 juvenile (<7 months old) animals and 1 adult cat (2 years old), that presented with dyspnea immediately after induction at local veterinary clinics and were referred to the Seoul National University Veterinary Medicine Teaching Hospital are described. Four animals were premedicated with atropine, and all were anesthetized intravenously using a combination of an alpha 2-adrenergic agonist (medetomidine or xylazine) and a dissociative anesthetic (ketamine or Zoletil^®^). Both dogs developed immediate epistaxis, dyspnea, and radiographic evidence of diffuse alveolar infiltration. One dog was euthanized after experiencing seizures. All 3 cats developed anorexia followed by dyspnea within 24 to 48 h post-anesthesia, resulting in death in 2 cats, while 1 cat recovered with symptomatic treatment. The sympathomimetic effects of dissociative anesthetics and vasoconstrictive alpha 2-adrenergic agonists can cause transient hypertension, which can precipitate pulmonary edema and hemorrhage, leading to dyspnea. Either juvenile or atropine-premedicated patients may be at an increased risk, warranting dose adjustment, route selection, and careful monitoring during anesthesia.

## Simple Summary

A combination of sedative and anesthetic drugs is commonly used to anesthetize dogs and cats for medical procedures. This report describes five young animals (two dogs and three cats) that developed serious breathing problems after receiving such a drug combination through injection into a vein. Most of the animals were less than seven months old, and four had also received a heart rate-stabilizing drug before anesthesia. Both dogs immediately developed nosebleeds and difficulty breathing, while all three cats showed delayed breathing problems within one to two days after the procedure. Despite treatment, two cats and one dog did not survive. The breathing difficulties were likely caused by a sudden rise in blood pressure from the drug combination, which forced fluid and blood into the lungs. These findings suggest that veterinarians should use lower drug doses in young animals, avoid unnecessary pre-anesthetic medications that may worsen blood pressure increases, and consider injecting the drugs into muscle rather than directly into a vein to reduce the risk of these potentially fatal side effects.

## 1. Introduction

Injectable anesthesia is widely used in small animal practice, particularly for short procedures and in relatively healthy patients [1]. It offers the practical advantage of being administered using only anesthetic agents and syringes, without requiring specialized anesthetic equipment or oxygen delivery systems, and reduces environmental contamination caused by anesthetic gases [2]. Injectable anesthetic agents can be administrated via intramuscular (IM) and intravenous (IV) routes. With the increasing use of combination anesthetics, the IV route is generally preferred because of its rapid induction and reduced injection pain, whereas the IM route is preferred because of its ease of administration.

An ideal injectable anesthetic should provide rapid onset, adequate muscle relaxation, analgesia, minimal cardiovascular depression, and predictable induction, maintenance, and recovery [2]. However, no single agent achieves all these features, making drug combinations a common strategy to enhance efficacy and reduce adverse effects. Dissociative anesthetics combined with alpha 2-adrenergic agonists are widely used in dogs and cats [3]. While dissociative anesthetics offer minimal cardiovascular depression, they pose risks such as a short duration of action, excessive salivation, muscle rigidity, and unstable recovery [3]. Co-administration of an alpha 2-agonist mitigates these effects and enables dose reduction, because its cardiovascular depression is dose-dependent. In South Korea, tiletamine/zolazepam and ketamine are commonly used dissociative anesthetics, and medetomidine and xylazine are routinely utilized as alpha 2-agonists in clinical practice. Although widely used, this drug combination has been reported to cause cardiovascular alterations, including bradycardia, decreased stroke volume, reduced cardiac output, and increased systemic vascular resistance [4].

This case series examined 5 animals (2 dogs and 3 cats) that developed dyspnea and pulmonary lesions following the intravenous administration of this combination, with a focusing on the administration route, age, and atropine use.

## 2. Case Description

Medical data were collected from local clinic records, owner histories, and the Seoul National University Veterinary Medical Teaching Hospital (SNU-VMTH) records (Table 1). Four of the animals were 5–7 months old, except for one cat that was 2 years and 2 months old. Pre-anesthetic blood chemistry was performed in 2 cats and was within the normal ranges. Dog 1 had intermittent vomiting and diarrhea, and whereas cat 1 had occasional coughing and ocular discharge. No additional abnormalities were noted during the pre-anesthetic physical examination in any case.

At the referring local veterinary clinics, atropine was intravenously administered as a routine premedication. Anesthesia was induced via IV administration of a combination of the dissociative anesthetic ketamine or Zoletil^®^, and the alpha 2-adrenergic agonist medetomidine or xylazine (Table 2).

**Figure 1:**
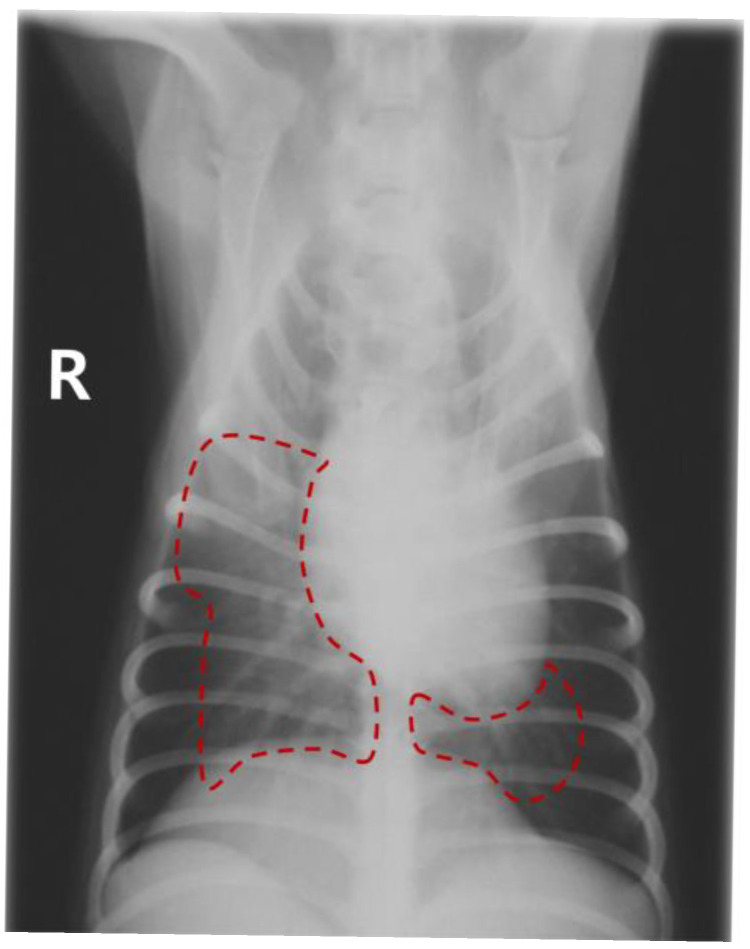
Ventrodorsal thoracic radiograph of Dog 1. Severe alveolar infiltration is present in the right middle and caudal and left caudal lung fields (outlined by the red dotted lines). The letter “R” indicates the right side of the patient.

**Figure 2:**
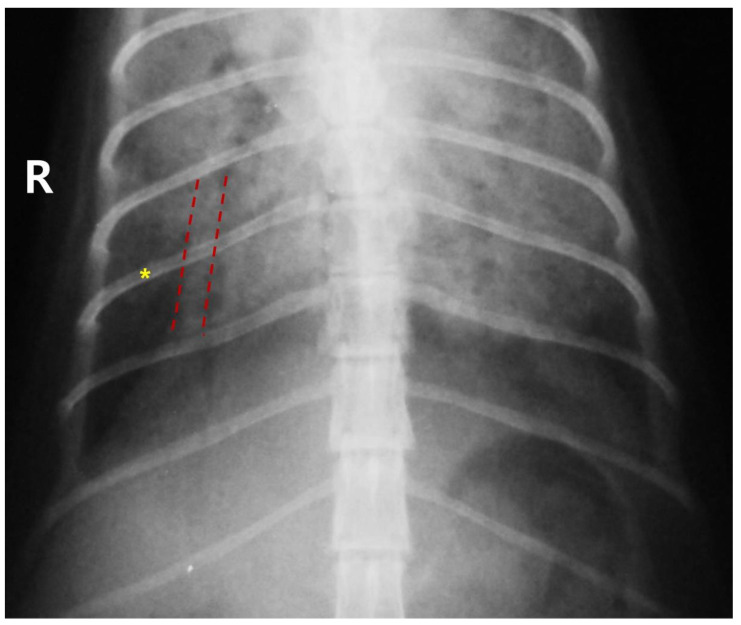
Thoracic radiograph of Cat 2 demonstrating pulmonary artery dilation. Ventrodorsal thoracic radiograph of Cat 2, showing dilation of the pulmonary artery (outlined by the red dotted line) and diffuse alveolar infiltration throughout the lung fields. The ninth rib is indicated by a yellow asterisk. The letter “R” indicates the right side of the patient.

Epistaxis and dyspnea occurred immediately after anesthetics administration in both dogs (Table 2). No abnormalities were observed during the induction or maintenance of anesthesia in any of the cats, and all recovered uneventfully. However, all 3 cats developed anorexia, followed by the onset of dyspnea within 24–48 h after their respective procedures. Dog 1 exhibited severe alveolar infiltration in the right middle and caudal and left caudal lung fields (Figure 1). Dog 2 showed severe alveolar infiltration in the right caudal and left cranial lung fields. Cat 1 presented with severe alveolar infiltration, leading to distortion of the cardiac silhouette and pulmonary artery dilation. Cats 2 and 3 exhibited diffuse alveolar infiltration and pulmonary artery dilation (Figure 2), which improved after 4 days.

Symptomatic treatment was provided at both local veterinary clinics and SNU-VMTH, including oxygen therapy, diuretics, corticosteroids, aminophylline, antibiotics, and alpha 2-antagonists as appropriate. Despite treatment, 2 cats died of progressive respiratory distress, and 1 dog developed seizures and was euthanized with the owner’s consent (Table 2). One dog and 1 cat recovered, requiring 2 weeks and 4 days, respectively.

## 3. Discussion

In this report, 2 dogs and 2 cats received IV atropine as premedication, followed by a combination of dissociative anesthetics and alpha 2-agonists administered via the same route. In both dogs, surgery could not be initiated owing to the onset of acute epistaxis immediately after anesthetics administration. All cats underwent an uneventful anesthetic procedure, including recovery. However, they subsequently experienced anorexia and lethargy, followed by the onset of dyspnea 24–48 h later. Thoracic radiography revealed diffuse alveolar infiltration throughout the lungs in all patients. Notably, all cats exhibited marked pulmonary artery dilation.

Alpha 2-agonists alone induce systemic vasoconstriction, including arterioles, leading to a transient but marked increase in systemic vascular resistance and blood pressure in dogs, typically lasting up to 20 min [5,6]. Concurrently, bradycardia occurs as a compensatory mechanism for hypertension, reducing the heart rate to less than 50% of its baseline [7]. This process can be predicted using the following equation [8]:

MAP (mean arterial pressure) = HR (heart rate) × SV (stroke volume) × SVR (systemic vascular resistance)

When an alpha 2-adrenergic agonist is combined with a dissociative anesthetic such as ketamine, compensatory bradycardia to initial hypertension may be inhibited, and the sympathomimetic effect of ketamine can cause tachycardia [9]. If IV atropine has been administered beforehand, its anticholinergic action may further augment the heart rate response, resulting in sustained hypertension and tachycardia during the early phase of anesthesia. In dogs, the acute clinical signs following anesthetic administration are presumed to result from transient increases in blood pressure and heart rate, leading to pulmonary hypertension and hemorrhage. The initial hypertensive response to alpha 2-adrenergic agonists is less pronounced in cats than in dogs [5]. This difference is likely attributable to drug-induced myocardial depression, which reduces stroke volume and lowers the risk of acute pulmonary hemorrhage in cats compared with dogs [10]. However, alpha 2-adrenergic agonists have been demonstrated to induce pulmonary vascular endothelial damage and increase microvascular permeability in experimental models [11,12]. Although these acute pulmonary responses are most pronounced in ruminants, subclinical microvascular injury may also occur in cats. A retrospective analysis of pharmacovigilance reports submitted to the Finnish Medicines Agency documented 61 cases of suspected pulmonary edema in 90 cats following alpha-adrenoceptor agonist administration, with onset ranging from 1 min to 2 days (median 15 min) [13]. The delayed onset of dyspnea in the present feline cases (24–48 h) may therefore reflect a secondary inflammatory cascade—including neutrophil recruitment and increased capillary permeability—triggered by initial subclinical microvascular damage during the acute hemodynamic insult, analogous to the delayed presentation observed in acute lung injury. The marked pulmonary artery dilation observed radiographically in all three cats further supports significant pulmonary vascular involvement beyond simple cardiogenic edema. However, without advanced diagnostics such as echocardiography, bronchoalveolar lavage, or serial cardiac biomarkers, the exact mechanism cannot be definitively determined.

Given these cardiovascular complications, readily available reversal options, such as alpha 2-antagonists, are clinically beneficial. Peripheral vasoconstriction, a well-documented adverse effect of alpha 2-adrenergic agonists, can be reversed by administering an alpha 2-antagonist, such as atipamezole [7]. Therefore, in young or geriatric patients, or when acute adverse effects arise, postoperative administration of an antagonist may facilitate recovery. However, because dissociative anesthetics are not reversed by alpha 2-antagonists, the overall quality of recovery may be suboptimal, and appropriate preparations should be made in advance.

In addition to species-specific differences, IV administration of anesthetic agents used in all cases in this report critically influenced their hemodynamic effects. Arteriolar vasoconstriction associated with alpha 2-adrenergic agonists occurs primarily when high doses are administered intravenously, whereas IM administration may mitigate this adverse effect [2].

The risk of these complications may be amplified in juvenile animals because of their physiological vulnerabilities. Of the 5 animals included, 4 were younger than 7 months of age. Owing to immature myocardial development, juvenile animals exhibit limited cardiac reserves and a relatively fixed stroke volume, which reduces their ability to compensate for hemodynamic changes such as increased peripheral vascular resistance [14]. In addition to cardiovascular limitations, juvenile animals have higher respiratory demands. Consequently, once dyspnea develops, clinical signs of hypoxemia may emerge more rapidly because of a reduced pulmonary reserve [14,15].

Atropine premedication may have adversely affected patient outcomes. In 4 of the 5 cases, atropine was administered at the referring clinics as a routine premedication prior to anesthesia to prevent bradycardia and arrhythmias, which are well-documented adverse effects of alpha 2-adrenergic agonists. However, the combination of intense peripheral vasoconstriction from alpha 2-agonists with atropine-induced prevention of compensatory bradycardia creates a predictable scenario of sustained hypertension and increased myocardial oxygen demand [2]. This concern is heightened when alpha 2-agonists are combined with dissociative anesthetics, as this combination may cause sustained tachycardia, further exacerbating myocardial dysfunction. Therefore, the use of atropine as a prophylactic premedication is not recommended when alpha 2-agonists are administered, particularly at high doses or via the intravenous route [2]. If anticholinergic therapy is deemed necessary, reactive administration—specifically when hemodynamically significant bradycardia with concurrent hypotension occurs during anesthesia—is preferable to prophylactic use. Glycopyrrolate, with its slower onset and more gradual heart rate response, may be a safer alternative in this context.

In addition to route-related considerations, the doses administered in this case series warrant critical evaluation. Notably, Cat 3 received medetomidine at 80 μg/kg intravenously; this dose falls within the published range for intramuscular administration in cats but substantially exceeds the recommended intravenous dose range (1–10 μg/kg in cats). Because IV administration provides immediate and complete systemic bioavailability without the gradual absorption phase inherent to intramuscular injection, applying IM-referenced doses via the IV route results in markedly higher peak plasma concentrations, effectively constituting a relative overdose. This distinction between IV and IM dose ranges is critical for safe clinical practice and should not be overlooked when selecting the administration route. Furthermore, the practical aspects of anesthetic preparation should be considered as a secondary risk factor. The residual volume in the syringe hub (approximately 0.1 mL) may lead to an unintentional additional overdose when drugs are combined in a single syringe, which is particularly critical for medetomidine in small patients (<5 kg), for which even a slight excess can be clinically significant [16].

This case series has several limitations that should be acknowledged. First, only five animals were included, and the anesthetic protocols were not uniform across cases, involving different drug combinations, doses, and premedication regimens. Therefore, a definitive causal relationship between the drug combination and the respiratory complications cannot be established, and these findings should be interpreted as hypothesis-generating observations. Second, the proposed pathophysiological mechanism remains presumptive, as intraoperative hemodynamic monitoring data (e.g., direct arterial blood pressure or cardiac output measurements) were not available from the referring clinics. Future prospective studies with standardized hemodynamic monitoring would be necessary to confirm the hypothesized mechanism. Third, pre-anesthetic blood work and baseline imaging were not performed in several cases; the absence of pre-anesthetic diagnostic evaluation should be recognized as an additional risk factor, particularly in juvenile patients.

## 4. Conclusions

The administration of a combination of dissociative anesthetics and alpha 2-adrenergic agonists may induce dyspnea as an adverse effect related to transient hypertension and pulmonary vascular injury. Multiple risk factors—including IV administration, young age, pre-anesthetic atropine use, and inappropriate dosing—may collectively increase the risk of respiratory complications. To minimize post-anesthetic complications, the following recommendations are proposed. First and most importantly, IV and IM dose ranges for alpha 2-adrenergic agonists are not interchangeable. Intramuscular administration should be the preferred route; when IV administration is unavoidable, doses must be substantially reduced (e.g., medetomidine 1–10 μg/kg IV in cats, compared with 10–80 μg/kg IM), as applying IM-referenced doses intravenously constitutes a relative overdose. Second, anesthetic agents should be drawn into separate syringes to avoid inadvertent overdose from syringe dead space, which is particularly significant in small patients (<5 kg). Third, prophylactic atropine administration should be avoided when alpha 2-agonists are used; anticholinergic therapy should be reserved for hemodynamically significant bradycardia with concurrent hypotension during anesthesia. Fourth, baseline diagnostic evaluation, including hematology, serum chemistry, and thoracic radiography, is recommended for juvenile patients undergoing injectable anesthesia.

## Figures and Tables

**Table 1:** Preanesthetic information about 2 dogs and 3 cats with dyspnea.

Parameter	Dog 1	Dog 2	Cat 1	Cat 2	Cat 3
Breed	Cocker Spaniel	Poodle	Korean Shorthair	Mixed	Turkish Angora
Sex	MC	M	MC	MC	FS
Age	5 ms	7 ms	2 ys 2 ms	5 ms	6 ms
Body Weight (kg)	4.5	2.0	—	3.3	—
Thoracic radiography	×	×	×	×	×
Blood analysis	×	×	Serum chemistry	×	Serum chemistry
Medical history	Intermittent vomiting and diarrhea	—	Intermittent cough and eye discharge	—	—

M: male; MC: castrated male; ms: months; FS: spayed female; ys: years; ×: not performed; —: not informed.

**Table 2:** Anesthetic drugs administered and clinical signs and results for 2 dogs and 3 cats presenting with dyspnea.

Drug (Concentration)	Route	Unit	Dog 1	Dog 2	Cat 1	Cat 2	Cat 3
Atropine (0.5 mg/mL)	IV	mg/kg	0.05	0.05	0.05	0.05	—
Ketamine (50 mg/mL)	IV	mg/kg	4	5	—	4	2.5
Zoletil^®^ (50 mg/mL)	IV	mg/kg	—	—	4	—	—
Medetomidine (1 mg/mL)	IV	μg/kg	30	—	20	15	80
Xylazine (20 mg/mL)	IV	mg/kg	—	2	—	—	—
Atipamezole (5 mg/mL)	IV	μg/kg	150	—	50	75	200
**Clinical Signs and Results**							
**Parameter**			**Dog 1**	**Dog 2**	**Cat 1**	**Cat 2**	**Cat 3**
Clinical signs			Epistaxis Dyspnea	Epistaxis Dyspnea	Anorexia Dyspnea	Anorexia Epistaxis Dyspnea	Anorexia Vomiting Dyspnea
Onset time			After injection	After injection	After 24 h	After 36 h	After 48 h
Result			Recovery (2 weeks)	Euthanasia	Death	Recovery (4 days)	Death

IV: intravenous; —: not performed.

## Data Availability

The data presented in this study are available on request from the corresponding author.
